# Supplementary material for: Illness perspectives in patients with primary aldosteronism
Source: PLoS One. 2022 Nov 28;17(11):e0277902. doi: 10.1371/journal.pone.0277902 (PMC9704575; doi:10.1371/journal.pone.0277902)
Supplement: Attachment — Submitted filename: Respons to reviewer.docx [file pone.0277902.s001.docx]

**Comments to the Author**

**Reviewer #1: The study of Ragnarsson O. and colleagues qualitatively describes the quality of life of patients with primary aldosteronism before, during and after treatment in both, unilateral and bilateral disease. This is the first study to adopt a qualitative approach to this topic and therefore is of particular interest.**

**The study is well designed and described; however, some issues are presents and are listed below:

Methods:
• I suggest to provide more information for screening test, confirmatory test and respective cut-offs for PA diagnosis.**

We have added this information to the revised version as suggested by the reviewer.

**• More details on AVS methods (stimulated or unstimulated? simultaneous or sequential?) criteria should be provided**

We have added this information to the revised version as suggested by the reviewer.

**Results:**

**• How many medically treated patients were treated with spironolactone? How many with eplerenone? Did the authors notice any difference between the two groups?**

Of 12 patients with bilateral disease, 10 were receiving eplerenone and two spironolactone. This information has been added to the method-section. Unfortunately, the low number of patients on spironolactone makes it impossible to compare the two groups.

**• There was any difference between patients that showed complete clinical success after adrenalectomy (defined according to PASO criteria, Williams TA, Lancet DE 2017 doi: 10.1016/
S2213-8587(17)30135-3) and those that showed partial or absent clinical success? Did biochemical success influence the satisfaction after adrenalectomy?**

Information on clinical and biochemical success in surgically treated patients, according to PASO, has been added to the method-section. The question whether there is a difference between patients with complete versus partial/absent clinical success is interesting. However, due to the relatively low number of patients (7 versus 6), this type of analysis cannot be performed in this study. We have, however, added a brief text to the discussion on this limitation.

**Discussion:**

**• Many studies reported that QOL is reduced in patients with PA, compared to the general population. However, patients with PA show similar QOL compared to patients with EH matched for hypertension severity (Buffolo F, EJCI 2021, DOI: 10.1111/eci.13419). This finding may suggest that hypertension and antihypertensive drug treatment may be the most important factors affecting QOL in PA patients, more than the effect of aldosterone per se. I suggest to briefly discuss this aspect in Discussion.**

We thank the reviewer for this comment. We have added a brief discussion on this important topic to the discussion.

**• Some authors reported that patients with PA showed reduced well-being and higher levels of stress and psychological distress (Sonino N, JCEM 2011; doi: 10.1210/jc.2010-2723). Moerover, depressive symptoms improve at FU after adrenalectomy (Citton, BMC surgery 2019, doi:10.1186/s12893-018-0432-1). I would discuss this themes in Discussion describing whether some of this aspects emerged from the interviews of this study.**

We appreciate this comment and have added discussion on this topics to the revised version of the manuscript.
